# Towards eHealth to support the health journey of headache patients: a scoping review

**DOI:** 10.1007/s00415-020-09981-3

**Published:** 2020-06-11

**Authors:** Daniëlle L. van de Graaf, Guus G. Schoonman, Mirela Habibović, Steffen C. Pauws

**Affiliations:** 1 Department of TSDH Research Center, Tilburg University, Tilburg, The Netherlands; 2 Department of Neurology, Elizabeth-TweeSteden Hospital, Tilburg, The Netherlands; 3 Department of Medical and Clinical Psychology, Tilburg University, Tilburg, The Netherlands; 4 Department of Cardiology, Elizabeth-TweeSteden Hospital, Tilburg, The Netherlands; 5 TiCC-Tilburg University, Tilburg, The Netherlands; 6 Philips Research, Healthcare, Eindhoven, The Netherlands

**Keywords:** Migraine, Tension-type headache, Cluster headache, Digital health tool, mHealth

## Abstract

**Objective:**

The aim of this study is to (1) review the digital health tools that have been used in headache studies, and (2) discuss the effectivity and reliability of these tools.

**Background:**

Many headache patients travel a long and troublesome journey from first symptoms until a meaningful care plan. eHealth, mHealth, and digital therapeutic modalities have been advocated as the way forward to improve patient care.

**Method:**

Online databases PubMed, Cinahl, and PsycINFO were searched using a predefined search query. A data extraction form was used to gather relevant data elements from the selected papers.

**Results:**

A total of 39 studies were selected. The studies included 94,127 participants. The majority of studies focused on diaries (*N* = 27 out of 39). Digital (cognitive) behavioral therapy were also quite common (*N* = 7 out of 39). Other digital health tool categories were tele-consultations, telemonitoring and patient portals.

**Conclusion:**

Many digital health tools for headache patients regarding diaries and behavioral/therapeutical treatment are described in scientific research with limited information on effectivity and reliability. Scientific knowledge with regard to other categories such as tele-consultations, patient portals, telemonitoring including medication adherence, online information resources, wearable, symptom checkers, digital peer support is still scarce or missing.

## Introduction

Headache is a common cause of disability. In a large population-based study of 16,256 people, approximately 20% reported experiencing headache on more than 5 days per month and 3.7% on more than 15 days per month [1]. Many headache patients travel a long and troublesome journey from first symptoms until a meaningful care plan including treatment. Ideally, a care plan should include a clear diagnosis, a treatment protocol, and a coaching strategy [2]. However, to come up with an effective care plan, important challenges that are associated with patients’ health journey must be overcome. These challenges include: (a) incorrect self-care by patient, (b) misdiagnosis or incomplete diagnosis by healthcare professionals, (c) waiting times in healthcare and doctor delays, (d) incorrect or inappropriate therapy, (d) under/over medication, (e) incorrect management of co-morbidities, (f) miscommunication between patient and specialists, (g) lack of guidance, and (h) misconceptions or misunderstandings from peers or work setting [3, 4].

With the pressing demand on the health care system, a shift towards the use of eHealth, mHealth, and digital therapeutic modalities has been advocated as the way forward to improve patient care and provide support to larger (underserved) groups of patients [5, 6]. We define eHealth as an abbreviation for electronic health to refer to the use of digital information and services in communication over the Internet to support and improve health or healthcare delivery to individuals. mHealth is the abbreviation for mobile health with refers to the use of mostly personal mobile or wearable electronic devices like smartphones, tablets, and wearables in health and healthcare delivery. If evidence-based treatment interventions are rendered to diagnosed patients via high-quality medically approved eHealth or mHealth software technology, we have reserved the specific but recent term of digital therapeutics or, short named, DTx [7]. As compared to traditional approaches, where patients have to visit the out-patient clinic for diagnosis and treatment, eHealth enabled care is about reducing the number of in-person visits while at the same time getting more (accurate) information from the patients via remote devices [8]. If properly validated on safety and outcome, digital form of consult and/or treatment could make care delivery safer, decrease patient burden, be more cost-efficient and more effective in patient outcome. For instance, within the mental healthcare, behavioral eHealth interventions have shown promising results which are comparable to traditional face-to-face treatment effects [9, 10]. In addition, studies also show that eHealth approaches are very well accepted by patients and could be implemented in the clinical practice [11]. A systematic review concluded that eHealth to remotely monitor patients with the long-term condition of heart failure reduced all-cause mortality by 34% and hospitalization by 21%, but that there are still mixed and heterogeneous effects for eHealth for heart failure across studies [12].

With respect to headache treatment and management, the use of eHealth applications or digital therapeutics has also been advocated. This has resulted in the development of mobile applications for diagnosis and disease management [13]. However, due to the many (fast) developments in eHealth, it is largely unknown which eHealth tools are available and, more importantly, also effective in the treatment of headache patients. Hence, the aim of this study is to (1) review the digital health tools that have been used in headache studies, and (2) discuss the effectivity and reliability of these tools.

## Method

### Search strategy

Online databases PubMed, Cinahl, and PsycINFO were searched to retrieve research studies concerning digital health tools for headache patients. In addition, hand search was performed screening the references of included articles and previous review paper. The search included English articles that were published between January 1st 2000 and January 1st 2019. The following main search terms were used: headache; migraine disorder; tension-type headache; cluster headache; headache disorder; telemedicine; patient portal; mobile applications; electronic diary; smartphone; and self-monitoring. Complete search strategy for each database is described in Table 1.

**Table 1:** Search strategy

Database	Keywords	Hits
Cinahl	((MH "Headache+") OR (TI headache) OR (AB headache) OR (TI headaches) OR (AB headaches) OR (MH "Migraine") OR (TI "migraine") OR (AB “migraine”) OR (TI "migraine") OR (AB “migraine”) OR (MH "Tension Headache") OR (MH "Cluster Headache")) ((MH "Telemedicine+") OR (MH "Telehealth+") OR (TI "telemedicine”) OR (AB “"telemedicine”) OR (TI "telehealth") OR (AB “telehealth”) OR (TI “telecare”) OR (AB “telecare”) OR (TI "mobile health") OR (AB "mobile health") OR (TI "mhealth") OR (AB "mhealth") OR (TI "ehealth") OR (AB "ehealth") OR (MH "Patient Portals") OR (TI "patient portal") OR (AB “patient portal") OR (TI "patient portals") OR (AB “patient portals") OR (MH "Mobile Applications") OR (TI “mobile application") OR (AB “mobile application") OR (TI “mobile applications") OR (AB “mobile applications") OR (TI app) OR (AB app) OR (TI apps) OR (AB apps)) ((TI “electronic diary”) OR (AB “electronic diary”) OR (TI “e-diary”) OR (AB “e-diary”) OR (TI “headache e-diary”) OR (AB “headache e-diary”) OR (TI “headache e-diaries”) OR (AB “headache e-diaries”) OR (TI “headache diary”) OR (AB “headache diary”) OR (TI “headache diaries”) OR (AB “headache diaries”) OR (TI “pain e-diary”) OR (AB “pain e-diary”) OR (TI “pain e-diaries”) OR (AB “pain e-diaries”) OR (TI “pain diary”) OR (AB “pain diary”) OR (TI “pain diaries”) OR (AB “pain diaries”) OR (MH "Smartphone") OR (TI "smartphone") OR (AB "smartphone") OR (TI "smartphones") OR (AB "smartphones") OR (TI “self-monitoring”) OR (AB “self-monitoring”)) S2 OR S3 S1 AND S4 S1 AND S4 Limiters—Published Date: 20,000,101–20,191,231 Narrow by Language:—English Search modes—Boolean/Phrase	273
PsycINFO	((DE "Headache" OR DE "Migraine Headache" OR DE "Muscle Contraction Headache") OR (TI headache) OR (AB headache) OR (TI headaches) OR (AB headaches) OR (MM "Migraine Headache") OR (TI "migraine disorder") OR (AB “migraine disorder”) OR (TI "migraine disorders") OR (AB “migraine disorders”) OR (MM "Muscle Contraction Headache")) ((MM "Telemedicine") OR (TI "telemedicine”) OR (AB “"telemedicine”) OR (TI "telehealth") OR (AB “telehealth”) OR (TI “telecare”) OR (AB “telecare”) OR (TI "mobile health") OR (AB "mobile health") OR (TI "mhealth") OR (AB "mhealth") OR (TI "ehealth") OR (AB "ehealth") OR (TI "patient portal") OR (AB “patient portal") OR (TI "patient portals") OR (AB “patient portals") OR (TI “mobile application") OR (AB “mobile application") OR (TI “mobile applications") OR (AB “mobile applications") OR (TI app) … ((TI “electronic diary”) OR (AB “electronic diary”) OR (TI “e-diary”) OR (AB “e-diary”) OR (TI “headache e-diary”) OR (AB “headache e-diary”) OR (TI “headache e-diaries”) OR (AB “headache e-diaries”) OR (TI “headache diary”) OR (AB “headache diary”) OR (TI “headache diaries”) OR (AB “headache diaries”) OR (TI “pain e-diary”) OR (AB “pain e-diary”) OR (TI “pain e-diaries”) OR (AB “pain e-diaries”) OR (TI “pain diary”) OR (AB “pain diary”) OR (TI “pain diaries”) OR (AB “pain diaries”) OR (MM "Cell … S2 OR S3 S1 AND S4 Limiters—Publication Year: 2000–2019 Expanders—Apply equivalent subjects Narrow by Language:—english Search modes—Boolean/Phrase	256
PubMed	((("headache"[MeSH Terms] OR headache[Title/Abstract] OR headaches[Title/Abstract]) OR ("migraine disorders"[MeSH Terms] OR "migraine disorder"[Title/Abstract] OR "migraine disorders"[Title/Abstract]) OR ("tension-type headache"[MeSH Terms] OR "tension-type headache"[Title/Abstract] OR "tension-type headaches"[Title/Abstract] OR "tension type headache"[Title/Abstract] OR "tension type headaches"[Title/Abstract]) OR ("cluster headache"[MeSH Terms] OR "cluster headache"[Title/Abstract] OR "cluster headaches"[Title/Abstract]) OR ("headache disorders"[MeSH Terms] OR "headache disorder"[Title/Abstract] OR "headache disorders"[Title/Abstract])) AND (("telemedicine"[MeSH Terms] OR "telemedicine"[Title/Abstract] OR "telehealth"[Title/Abstract] OR "telecare"[Title/Abstract] OR "mobile health"[Title/Abstract] OR "mhealth"[Title/Abstract] OR "ehealth"[Title/Abstract]) OR ("patient portals"[MeSH Terms] OR "patient portal"[Title/Abstract] OR "patient portals"[Title/Abstract]) OR ("mobile applications"[MeSH Terms] OR "mobile application"[Title/Abstract] OR "mobile applications"[Title/Abstract] OR app[Title/Abstract] OR apps[Title/Abstract]) OR ("electronic diary"[Title/Abstract] OR "e-diary"[Title/Abstract] OR "headache e-diaries"[Title/Abstract] OR "headache diary"[Title/Abstract] OR "headache diaries"[Title/Abstract] OR "pain diary"[Title/Abstract] OR "pain diaries"[Title/Abstract]) OR ("smartphone"[MeSH Terms] OR "smartphone"[Title/Abstract] OR "smartphones"[Title/Abstract]) OR "self-monitoring"[Title/Abstract])) AND (("2000/01/01"[PDAT]: "3000/12/31"[PDAT]) AND (Dutch[lang] OR English[lang]))	440

### Study selection

**Fig. 1:**
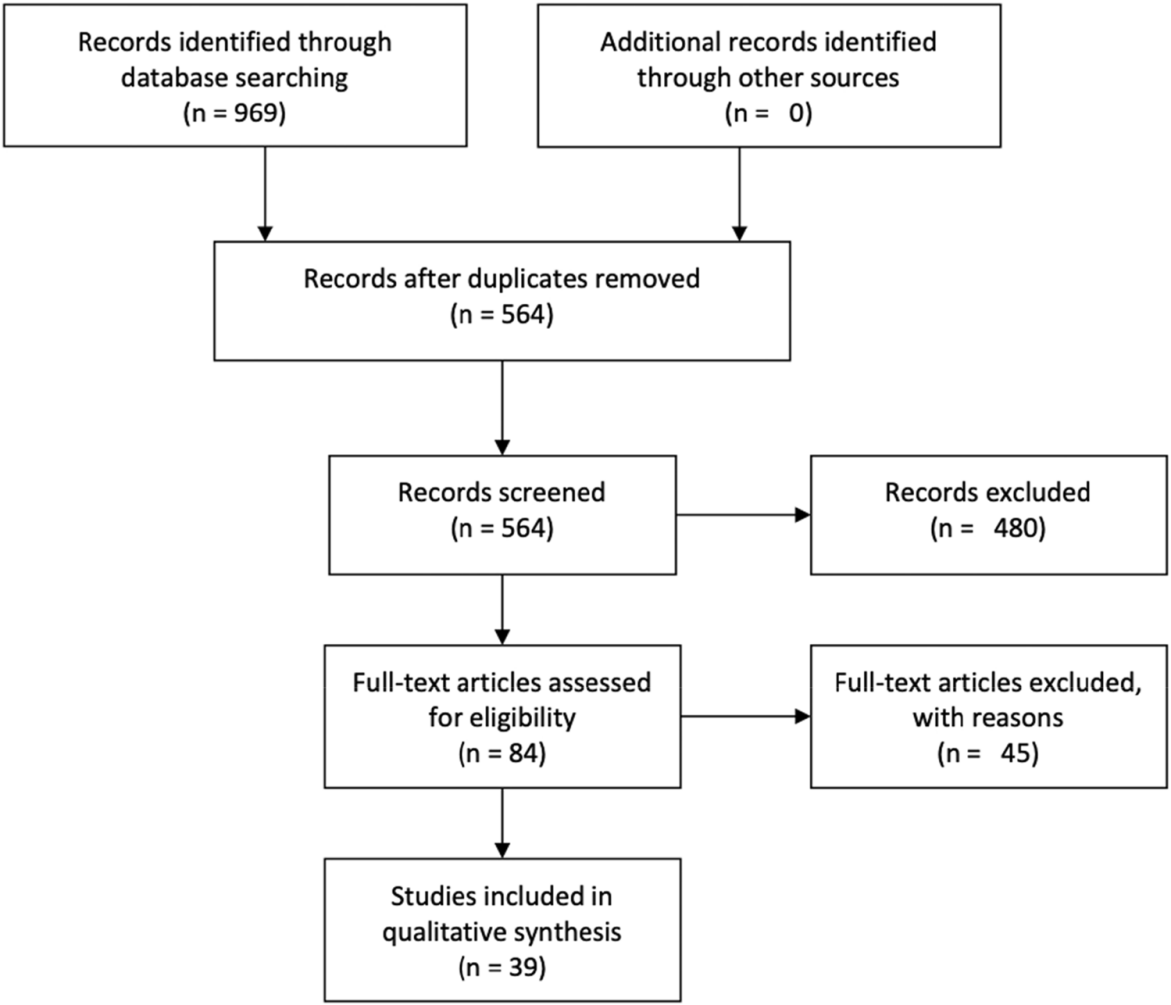
Flow diagram

In total, the search resulted in 969 studies (see Fig. 1). The complete search was then imported into EndNote to remove duplicates. Studies were included if the digital health tool related to measuring or monitoring (e.g. e-diary), meaning (e.g. information provision), or mediating (e.g. communicate with healthcare professional). Next to that, the explanation of the digital health tool had to be a full-length paragraph dealing with an explanatory description of the digital health tool to depict a complete, clear picture of the tool for this review. Studies regarding secondary headache disorders or interventions by telephone were excluded. Two authors (DG and GS) screened all articles as studies independently based on title and abstract using Covidence and made a selection of articles for full text screening. Full texts were screened independently by all four authors. The authors screened half (50%) of the studies while ensuring that any single study was screened by two authors; any pair of authors had 25% overlap in screened studies. During the complete selection process, discrepancies were discussed and subsequently resolved in planned follow-up team meetings. Articles that involved a review were not included in this study, though their referenced articles were checked for presence in our search results, and hand-selected if absent.

### Data extraction

Data extraction was performed by one of the authors (DG) using IBM SPSS Statistics 23 for tabulating the descriptive data from all articles. The following information categories were used from each study: digital health tool category, author, year, country/countries, tool name, operating system, manufacturer/vendor/developer, description of the tool, study design, participant characteristics in studies (sample size, age, and gender), headache type, and primary outcome.

## Results

A total of 39 studies were selected to be included in the review. The extracted data is shown in Table 2. Included articles were published within a time frame of 15 years (2003–2018). Up to and including 2014, one or two articles per year appeared to be relevant to our scoping purpose and therefore included in this review. Since 2015, the number of studies related to digital health tools for headache patients increased. The period from 2015 to 2018 amounted to a total of 26 studies. In sum, the studies included 94,127 participants. One study did not report the number of participants. The majority of studies mainly had female participants. In four studies, males were not included at all. For 20 studies, participants were adults only. Four studies only included children and eight studies included both children and adults. Studies mainly focused on migraine (*N* = 29 out of 39), cluster headache (*N* = 14 out of 39), and tension-type headache (*N* = 8 out of 39), concurrently or independently. Other types of headache were examined by 25 studies, including medication-overuse headache and non-acute headache. The included studies in the search mainly entailed observational prospective cohort studies (*N* = 15 out of 39) and parallel-group randomized controlled trials (*N* = 12 out of 39). A summative usability evaluation was reported in four studies and an observational retrospective cohort study was included in three studies. Two articles included a formative usability evaluation (pilot) study. Qualitative formative usability study, single case clinical (or AB – Baseline-Intervention) study, and (web) development of an electronic diary were all conducted only once.

**Table 2:** Study characteristics

Digital health tool category	Author (reference), year (country/countries)	Tool name (operating system), manufacturer/vendor/developer	Description of tool	Study design	Participants: sample size, (age range or SD), females and males	Headache type (migraine, cluster, tension or other)	Primary outcome
Diary keeping	Allena et al. [32], 2012 (Italy)	Tool name unknown, (Windows CE version 3.0 operating system), ATC Service, Pavia, Italy	Palm device with diary and personal digital assistant	Parallel-group randomized clinical trial with usual care control to assess the effect of a doctor visit preparatory website on migraine management as intervention (myexpertdoctor.com) on doctor-patient communication	85 participants (age unknown), 68 females and 17 males	Other: medication-overuse headache	Participants found the electronic diary easy to understand and use. Compliance was higher compared to traditional paper version
	Bandarian-Balooch et al. [33], 2017 (Australia)	https://www.enhance diary.com (iOS, Android, Windows and website version using HTML)	Electronic diary	Parallel-group randomized controlled trial to examine differences in reliability, validity, and headache outcomes between e-diaries versus paper diaries	181 participants (18–55 year of age), 146 females and 35 males	Migraine, tension-type and other: migraine with aura and comorbid tension-type headache, migraine without aura and comorbid tension-type headache, probable migraine or tension-type headache	E-diaries and short-paper diaries resulted in less missing data and errors than long-paper diaries. Also, e-diaries and short-paper diaries were less heavy and less difficult to use than long-paper diaries
	Barmettler et al. [34], 2015 (USA)	REDCap (Safari, Internet Explorer, Chrome)	Record and track temporal pattern of migraine	Observational prospective cohort study to assess pain onset, location and distribution in patients with migraine	36 participants (20–55 years of age), 28 females and 8 males	Migraine	The electronic tool for collecting data on the migraine headache patterns was able to capture patterns of pain distribution. It may also be used to determine changes in patterns of pain distribution
	Berengueres and Cadiou [35], 2016 (Singapore)	Migraine Buddy (Android, iOS), Healint	Electronic diary to monitor migraine episodes	Observational retrospective cohort study to assess triggers, symptoms and treatment in patients with migraine/Poor paper	83.000 participants (18–65 years of age), 72.920 females and 10.080 males	Migraine	Main triggers of migraine were stress, lack of sleep, and anxiety. Main symptoms of migraine were light sensitivity, noise, and neck pain. Main reliefs of migraine were sleep, dark room rest, and stay indoors (drugs not included)
	Cronin et al. [36], 2018 (Italy)	HeadApp!©	Mobile application for self-reporting	Observational retrospective cohort study for algorithm construction/Non-clinical	1194 participants, age and gender unknown	Migraine	Self-reported data from mobile applications can be used to construct algorithms for identifying migraine criteria and medication, such as headache classification, usage and effectiveness
	Filipi and Khairat [37], 2013 (USA)	Tool name unknown (web-based)	Web-based diary. Full-size website and mobile website	(Web) Development of a e-diary/This paper does not report on a study	Unknown	Other: chronic headache	A full-size website and mobile website were designed and implemented, by which users can input data about their headaches
	Giffin et al. [38], 2003 (UK)	Philips Nino Model 312 (Microsoft Windows CE), Philips Electronics N.V., New York, NY/Provenda Biometrics, Inc., Blue Bell, PA (customization)	Device with diary questions to monitor symptoms of attacks	Observational prospective (multi-center) cohort study to assess non-headache symptoms before, during and after migraine attacks in patients with migraine	97 participants (24–69 years of age), 92 females and 5 males	Migraine	Main premonitory symptoms were fatigue, having difficulty concentrating, and stiff neck
	Giffin et al. [39], 2016 (UK)	Tool name unknown	Electronic diary with daily alarming including symptom related questions	Observational prospective cohort study to examine migraine postdrome symptoms	120 participants, age and gender unknown	Migraine	Postdrome symptoms are common characteristics of migraine attacks for people who report non-headache symptoms
	Goldberg et al. [40], 2007 (USA)	Nino handheld device, Provenda Biometrics (Blue Bell, PA)	Mobile device with daily self-assessment (including alarming)	Observational prospective cohort study to evaluate an electronic diary tool to evaluate migraine during menstrual period and relationship of headache to menstrual symptoms	20 participants (21–47 years of age), 20 females	Migraine and other: headaches and premenstrual symptoms	The electronic diary led to many abnormal endings of sessions. It was not clear whether this was because of the subject or device error
	Heyer and Rose [19], 2015 (USA)	Tool name unknown	Electronic diary	Observational prospective (longitudinal) cohort study to assess compliance with a diary protocol in patients with migraine	52 participants (10–18 years of age), 38 females and 14 males	Migraine	Compliance was highest on days when abortive headache medicine was used or in the first 2 weeks of the diary. Compliance was lowest on evenings
	Houle et al. [41], 2017 (USA)	Palm Pilot with Pendragon forms software and REDCap software (iOS and web-based), Pendragon and REDCap	Diary assessing headache activity, characteristics and medication use	Observational prospective (longitudinal) cohort study to develop a forecasting model for headache	95 participants (age unknown), 86 females and 9 males	Migraine and other: episodic migraine	Participants had headache attacks on 38.5% of all days. Through a prediction model, headache attacks can be predicted for different individuals
	Huguet et al. [42], 2015 (Canada)	myWireless Headache Intervention diary (myWHI diary) (iOS)	Electronic diary via mobile phone	Summative usability evaluation to assess usability principles of an e-diary/Psychometirc validation of diary items/this article reports on various studies at the same time	108 participants (14–28 years of age), 93 females and 15 males	Migraine and tension-type	Mobile diary was perceived as useful, easy to learn, and efficient to use
	Kikuchi et al. [43], 2012 (Japan)	Ruputer ECOLOG, Seiko Instruments Inc	Electronic diary	Observational prospective cohort study to assess headache intensity and exacerbations in patients with tension-type headache	31 participants (20–60 year of age), 22 females and 9 males	Tension-type	Tension-type headaches have significant diurnal variation, with the highest intensity in the late afternoon and the lowest intensity in the morning
	Kikuchi et al. [44], 2015 (Japan)	Ruputer ECOLOG (W-PS-DOS version 1.16), Seiko Instruments Inc., Tokyo, Japan	Electronic diary via watch-type computer	Observational prospective cohort study to asses pain intensity and pain interference in women with migraine and obesity	23 participants (20–59 years of age), 18 females and 5 males	Tension-type	Subsequent increase in headache exacerbation within 3 h was correlated to momentary psychological stress. This correlation was not found for the individual mean of psychological stress
	Kroon Van Diest et al. [45], 2016 (USA)	iMigraine Application 1.1 AND MEMS6® TrackCap (iOS), the Divisions of Behavioral Medicine and Clinical Psychology and Bioinformatics at CCHMC AND AARDEX Corporation	Medication adherence monitor and personal electronic diary device	Observational prospective cohort study to collect data on medication and lifestyle recommendation adherence in adolescents with migraine	56 participants (11–17 years of age), 40 females and 16 males	Migraine	Electronic monitoring resulted in higher rates of medication adherences compared to self-reported rates. This was higher for patients taking medication once a day compared to patients taking medication twice a day
	Moloney et al. [46], 2009 (USA)	Tool name unknown, (Javascript and Cold Fusion)	P&P headache diary and health history questionnaire. Electronic diary via personal digital assistant	Observational prospective cohort study to collect data via ecological momentary assessment in patients with tension-type headache to relate psychological stress to TTH exacerbations	77 participants (18–55 years of age), 77 females	Migraine	Personal digital assistant diaries resulted in higher data accuracy and feasibility than paper and pencil diaries
	Palermo et al. [47], 2004 (USA)	Hewlett Packard Jornada 548 (Windows CE version 3.0)	Electronic diary on a handheld computer	Parallel-group randomized controlled trial to assess impact on compliance, accuracy, and acceptability in e-diary versus p-diary	60 participants (8–16 years of age), 42 females and 18 males	Other: headache (or juvenile idiopathic arthritis)	Compliance was higher for children with e-diaries compared to p-diaries. Acceptability and ease-of-use were both equally high
	Park et al. [48], 2016 (Taiwan)	Tool name unknown, M2Community Co., Ltd	Smartphone Headache Diary Application (SHD). Monitor details concerning headache trigger factors and characteristics	Observational prospective cohort study to assess migraine triggers in episodic migraineurs	62 participants (19–55 years of age), 51 females and 11 males	Migraine	Smartphone Headache Diary Applications is complete in estimating episodic migraine triggers. Main triggers of migraine are stress, tiredness, and sleep deprivation. Risks of migraine can be increased by traveling, hormones, noise, alcohol, overeating, and stress
	Park et al., 2018 [49] (Korea)	Tool name unknown, M2Community Co., Ltd	Smartphone Headache Diary Application (SHD). Monitor headache trigger factors and characteristics	Observational prospective cohort study to assess circadian variations in the clinical presentation of migraine through a smartphone headache diary	82 participants (adults), 69 females and 13 males	Migraine	Headaches mostly occurred in the morning. Most headaches were non-migraine. In the morning, headache characteristics were most common
	Seng et al. [50], 2018 (USA)	N1-Headache® (Curelator Headache®) (iOS), Curelator Inc	Mobile headache diary (via physician, paid or free)	Observational prospective (longitudinal) cohort study to assess factors related to adherence with mobile headache diaries	1561 participants (18–80 year of age), 1376 females and 336 males	Migraine	Headache people have difficulties with adherence to electronic headache diaries. Lower adherence is associated with higher daily anxiety, younger age and free availability of the app
	Sorbi et al. [51], 2007 (The Netherlands)	PalmOne Treo 600 (Linux, supported by the software components Apache Web Server, Java, PostgresSQL, and Tomcat, and data encrypting is authorized by SSL certification), Palm Inc, Sunnyvale, CA, USA	Personal digital assistant for home-based cognitive-behavioral treatment. Focused on preventing attacks, identifying attacks and supportive preventive healthy behavior	Formative usability evaluation (pilot) study on online digital assistance in migraine/non-clinical	5 participants (24–52 years of age), 5 females	Migraine	Online digital assistance was acceptable, in terms of user-friendliness, absence of burden, and perceived support. Also, it was feasible, in terms of acceptable data loss and good participant compliance
	Tassorelli et al. [52] 2017 (Italy)	The Comoestas tool, COMOESTAS Project	Electronic diary including an alerting system and patient-doctor communication	Parallel-group randomized controlled trial to assess headache outcomes between Comoestas group (electronic monitoring) and the classic group (paper headache diary)	663 participants (18–65 years of age), 521 females and 142 males	Other: medication-overuse headache	Use of the electronic tool reduced medication overuse and improved adherence to treatment
	Thomas et al. [53], 2016 (USA)	Tool name unknown	Ecological momentary assessment based on smartphone	Observational prospective cohort study to asses pain intensity and pain interference in women with migraine and obesity	116 participants (18–50 year of age), 116 females	Migraine	Pain intensity appeared to be a determiner of pain interference on migraine headache days. Allodynia, pain catastrophizing and headache management self-efficacy are moderators of pain intensity
	Vo et al. [54], 2018	Migraine Buddy©	Smartphone application for self-reporting of migraine patterns, characteristics and mechanisms	Observational retrospective cohort study to assess burdens of migraine using a smartphone application	3900 participants (18–74 year of age), 2545 females and 336 males	Migraine	Migraine attacks strongly influence patients' health-related quality of life, work and personal well-being
Behavioral or therapeutical intervention	Huguet et al. [55], 2014 (Canada)	Tool name unknown (iOS)	iPhone interface for psychosocial treatment program	Qualitative formative usability study by means of a focus group of with prospective end users to identify user needs and preferences for ICT-mediated psychosocial support for headache self-care/Non-clinical	25 participants (14–28 year of age), 19 females and 6 males	Migraine and tension-type	According to participants, the smartphone pain diary should be used daily, must be easily accessible, should be customizable and interactive. Also, participants want to communicate with other headache patients and experts
	Kleiboer et al. [56], 2009 (The Netherlands)	PalmOne Treo 600 (Linux, supported by the software components Apache Web Server, Java, PostgresSQL, and Tomcat, and data encrypting is authorized by SSL certification), Palm Inc, Sunnyvale, CA, USA	Personal digital assistant (PDA) for cognitive-behavioral treatment. Home-based training of behavioral attack prevention and identifying attacks	Summative usability evaluation to assess acceptance of online self-management training for migraine by means of a survey to new patients and expert patients/non-clinical	44 participants (25–63 years of age), gender unknown	Migraine	Feasibility and acceptability were positively influenced by online digital assistance as part of behavioral training. However, online digital assistance as part of behavioral training did not result in more physical improvements compared to behavioral training only
	Law et al. [57], 2015 (USA)	Tool name unknown (Web-MAP)	Online cognitive-behavioral treatment for families. Web-Based Management of Adolescent Pain	Parallel-group randomized controlled trial to determine feasibility and effectiveness of internet cognitive-behavioral intervention adjunctive to specialized headache treatment versus specialized headache treatment alone	83 participants (11–17 years of age), 68 females and 15 males	Migraine and tension-type	For both Internet cognitive-behavioral group and specialized headache treatment group, a reduction in headache days was reported. However, there was no significant difference between those groups
	Sorbi et al. [14], 2015 (The Netherlands)	MyMigraine/ID MigraineTM SCL-90 R	Training in relaxation and cognitive-behavioral techniques. Eight lessons with homework	Parallel-group randomized controlled trial to examine effectiveness of online behavioral training in self-management versus waitlist control for migraine patients	368 participants (18–65 years of age), 314 females and 54 males	Migraine	Self-efficacy, internal and external control in migraine management, and migraine-specific quality of life only improved in online behavioral training and not in the waitlist control group
	Sorbi et al. [58], 2017 (The Netherlands)	MyMigraine/ID MigraineTM SCL-90 R	Training in relaxation and cognitive-behavioral techniques. Eight lessons with homework	Parallel-group randomized controlled trial to examine benefits of online behavioral training between receiving this directly or after 10 months of watchful training	468 participants (18–65 years of age), 406 females and 62 males	Migraine	Online behavioral therapy positively changed migraine frequency compared to 'watchful waiting'
	Sorbi and Van der Vaart [59], 2010 (The Netherlands)	MyMigraine	Self-management via internet training tool to prevent attacks	Summative usability evaluation to assess usability and feasibility principles and psychometrical soundness of an electronic headache diary	10 participants (33–68 years of age), 8 females and 2 males	Migraine	Study 1 included ratings of clarity, instructiveness, importance and ease of an Internet training aid, which were all rated positively. Study 2 included ratings of the web application, digital support, and web adaption of the protocol, which were also all rated positively
Diary keeping AND behavioral or therapeutical intervention	Devineni and Blanchard [60], 2005 (USA)	Tool name unknown (commercial Windows NT server with a Web address assigned to the University at Albany domain)	Database-backed study website. Internet technology including behavioral interventions and headache diary	Parallel-group randomized controlled trial to examine differences in pain symptoms and functional impairment in Internet-delivered treatment versus symptom monitoring alone	86 participants (adults), 71 females and 15 males	Migraine and tension-type	Internet-based treatment decreased headache activity, general headache symptoms, and headache-related disability compared to monitoring waitlist control. It was also more time saving
	Minen et al. [61], 2018 (USA)	RELAXaHEAD Application	Headache diary and program for progressive muscle relaxation	Formative usability evaluation of an app by thinking aloud protocol/Non-clinical	10 participants (20–74 years of age), 8 females and 2 males	Other: headache	Daily diary was reported as easy to use and understand, relevant for tracking headaches and relevant to personal interest and attention. The progressive muscle relaxation matched their interest and attention, but also improved their stress and low mood
Other: teleconsultation	Müller et al. [62], 2016 (Norway)	Tool name unknown, other: teleconsultation (Cisco C40 integrator package, Cisco C40 Integrator Multisite, Cisco Precision HD 1080p 12xcamera, NEC X551s 55″ LED monitor, Audio-Technica ceiling microphones and JBL LSR2325P active speakers, Integrator Package C40 dual display option, and Cisco touch-control device for C Series), Cisco	Telemedicine consultations via video conferencing system	Parallel-group randomized controlled trial to determine the differences in acceptability, feasibility, and costs between specialist telemedicine visits versus traditional specialist visits for headache patients	402 participants (16–65 years of age), gender unknown	Migraine, tension-type, other: trigeminal autonomic cephalalgia, new daily persistent headache, primary stabbing headache, medication-overuse headache	Telemedicine for non-acute headaches is another option to traditional consultations, which is accepted, feasible, time-saving, and cost-saving
	Müller et al. [63], 2017 (Norway)	Cisco C40 integrator package/Cisco EX60 (Cisco), Cisco	Telemedicine consultations	Parallel-group randomized controlled trial to determine long-term treatment efficacy and safety via telemedicine consultations versus traditional consultations	402 participants (16–65 years of age), 301 females and 101 males	Other: non-acute, and less likely secondary	No differences were found between telemedicine and traditional consultations in long-term treatment efficacy and safety
	Müller et al. [64], 2018 (Norway)	Tool name unknown	Telemedicine consultation via two-way audio and video	Parallel-group randomized controlled trial to determine patients satisfaction of telemedicine consultations and traditional consultations	402 participants (16–65 years of age), 301 females and 101 males	Other: non-acute headache	Long-term satisfaction was higher for telemedicine patients than for traditional consultations
	Qubty et al. [65], 2018 (USA)	Cisco WebEx application (Cisco), Cisco	Telemedicine consultations via laptop, desktop, tablet, or mobile phone	Summative usability evaluation by means of a survey to end users/non-clinical	51 participants (4–20 years of age), 93 females and 15 males	Other: headache	Patients and families are convinced that telemedicine is more convenient, causes less disruption of daily routine and is more cost-effective than clinic visit
Other: patient portal	Sciamanna et al. [66], 2006 (USA)	https://www.myexpertdoctor.com	Web-based computer program with patient-doctor communication. Patient receives personalized feedback, which can include questions the patient should consider, explanations, or referral to further information	Parallel-group randomized controlled trial to determine effects of using a migraine-specific doctor-patient communications website. Differences between using the website before (intervention) or after a visit (control) were assessed	50 participants (age unknown), 46 females and 4 males	Migraine	Web-based computer program (website) may positively influence doctor-patient communications, in terms of improved care and quality of life
Other: training tool for specialists	Raieli et al. [67], 2018 (Italy)	Tool name unknown, Janssen	Training tool for specialists, including different information sources, discussion page and WhatsApp group	Observational prospective cohort time-series (longitudinally—4 months) study to collect headache data via online diary to assess feasibility and acceptance properties of online diaries/non-clinical	67 participants, age and gender unknown	Migraine	Subscriptions were increased with about 80%, which shows there is an increased appreciation of social networks. Activity has not significantly increased
Other: telemonitoring (medication adherence)	Ramsey et al. [68], 2018 (USA)	MedaCheck app (www. medacheck.com) (Android, iOS)	Mobile phone reminder system for adherence	A single case clinical (or AB – Baseline-Intervention) study in which a series of measurements is taken repeatedly for individuals with different levels of intervention. Participant in study acts as his own control. Aim is to assess an app with reminder on adherence of preventive treatment in AYA with migraine	35 participants (13–21 years of age), 27 females and 8 males	Migraine	Mobile phone application adherence rates are significantly higher than self-reported app-based adherence rates. Also, acceptability and convenience were rated as high

### Digital health tool characteristics

All studies were classified among different digital health tool categories (see Table 2). The majority of studies focused on diaries (*N* = 27 out of 39). These diaries are being used to gather insight in symptom frequency and severity, keep track of medication use and provide a disease burden overview to the care provider. Digital (cognitive) behavioral therapy were also quite common (*N* = 7 out of 39). Other digital health tool categories were tele-consultations (*N* = 4 out of 39), telemonitoring (medication adherence) (*N* = 2 out of 39) and patient portal (patient-doctor) (*N* = 2 out of 39). One study examined a headache training tool for specialists. Tool names were not mentioned in 16 studies. In 17 studies, the operating platform was not mentioned. iOS was the main operating system as a platform that was most often used (*N* = 8 out of 39). Android and Windows CE were both mentioned in three articles as operating system. Other operating platforms were among others a Cisco proprietary platform, a Web-based platform (HTML), and a Java-based platform. Platform use is timeline specific. Internet platform technologies continue to change from years to years.

### Potential patient benefits of digital health tools for headache

Current search revealed that one of the most prominent eHealth tools used by patients with headache is an electronic headache diary. According to the evaluated studies, using an electronic headache diary seems to contribute to a more clear diagnosis, better assessment of headache burden, and accurate medication use and therapy response. Besides being a device for measurement, the use of electronic diaries also showed benefits in reducing medication overuse and improving medication adherence, identification of triggers or premonitory symptoms, the timing of headache attacks, the perceived pain during headache attacks, the main headache reliefs, and the impact of headache attacks on perceived quality of life (Table 2).

Besides headache diaries, a significant number of studies (*N* = 7 out of 39) has reported the results of online behavioral interventions aiming to reduce the headache burden. Relaxation training and cognitive-behavioral therapy have been evaluated as behavioral modalities and showed limited benefit. The use of an online relaxation or cognitive-behavioral training program showed a reduction in headache attack frequency or symptoms in comparison to ‘watchful monitoring’ alone, while its benefit as an adjunctive digital therapy to a specialized headache treatment is still not demonstrated. In addition, in their study, Sorbi et al. [14] showed that self-efficacy, control of migraine management, and migraine related quality of life increased in the intervention group as compared to the waiting list controls (Table 2).

Tele-consultation is the third major area of digital tools used in headache therapy. Tele-consultations are seen as non-inferior (or equivalent) to traditional consultations in safety and efficacy, but are thought to be more convenient and more cost-effective by patients and can positively influence patient-doctor communication (Table 2).

## Discussion

This scoping review aimed to survey available digital health tools for headache patients that can be used to gather insights into the disease, measure symptoms’ severity, and facilitate communication with health professionals. Our search revealed that most studies investigated headache diaries (*N* = 27) or online (cognitive) behavioral therapy (*N* = 7). Only a few studies looked at tele-consultations, patient portals, and telemonitoring including medication adherence. Based on the findings from these studies it can be concluded that electronic headache diaries are a must-have in the treatment delivery to patients with headache, both as an essential self-reported outcome measurement device. Only via daily self-reports, treatment options can detail out true patient benefits in timed events, location, signs and symptoms. Additionally, sincere self-report on lifestyle can help in identifying behaviors that might act as a cause or precursor in prodromal headache exacerbation stages like medication overuse and stress. However, diary adherence is a problem; diaries need to be improved in learnability and ease-of-use. Electronic diaries, as part of digital therapeutics proposition to patients, require multi-center clinical studies with patient cohorts to assess the effectiveness and truth worthiness of what will be self-reported. Online cognitive-behavioral therapy (CBT) is the foremost example of a digital therapeutics solution that could be applied to patients with headache complaints [10]. However, clinical supervision and professional improvement will further extend the potential relief and health benefits to patients.

The current review identified 27 different studies. In a review from 2014 a total of 38 different headache apps were identified [13] and at the time of writing this review the authors identified more than 100 headache apps in the Google play store only. According to a recent study [15], there are more than 120,000 mobile medical apps available in the various App Stores and across mobile platforms. Headache app can support the health journey of headache patients in several ways like providing digital headache education, diary keeping, classifying headache attacks, and keeping track of medication adherence. Some apps have more advanced features for measuring daily occurrence of a long list of trigger factors in combination with headache symptoms providing a detailed report on potential personal triggering mechanisms [16]. Data collected by means of diaries can be used in predicting the occurrence of headache attacks allowing for pro-active self-management [17, 18]. However, a large portion of people with headache finds difficulties in adhering to keeping a diary [19, 20]. Higher level of daily anxiety, younger age and free availability of the diary tool are associated with poorer adherence [20]. Apps can also be used to capture medical information in randomized clinical trials; they are then used as a measuring instrument to support the purpose of the trial [21]. We observed that many apps (> 80%) have been created without early or long-standing involvement of patients, medical professionals or representatives of the scientific community [13]. A recent review on medication apps showed that 15% of apps is produced as a co-creation effort with health care providers and 2.1% in collaboration with academia [22]. If health apps are considered medical devices dependent on their intended medical purpose of use, they need to be assessed or even certified on their usability, usefulness, patient safety, credibility and health effect, similar to pharmaceutical therapies [23]. It is therefore reassuring that the EU has formed national working groups to align and prepare for EU guidelines on ‘good and safe’ health apps. In addition, trustworthy institutions such as the National Health Service provide online facilities to recommend well-functioning health apps. Headache apps should be scrutinized on their safety and usability properties as well.

The second category of eHealth tools that we identified focuses on digital (cognitive) behavioral therapy. Studies showed that behavioral treatment is acceptable and feasible [24] in this population. However, the number of randomized controlled trials evaluating the effectiveness of behavioral interventions for patients with headache is relatively small and show mixed findings. This advocates for future studies to focus on development and evaluation of these interventions as previous studies in other patient samples have shown very promising results [25]. For patients suffering from headache online behavioral treatment could focus on specific triggers that have been associated with headache (e.g. distress, lifestyle, medication adherence). Previous studies have shown that online behavioral treatment can be as effective as face-to-face treatment in reducing distress [9, 10]. In addition, it might be a cost-effective way tor reach large proportions of the targeted population [26]. However, studies do show that online treatment is more likely to be effective when blended with personal contact between patient and therapist [27]. A fully automated treatment is less likely to be effective as ‘one size fits all’ approach seems not to be the way forward [28]. Hence, future studies should focus on, blended care, personalized interventions that appeal to patients’ needs and preferences, but are at the same time scalable.

The third category includes a few studies on patient portals, tele-consultations and telemonitoring including medication adherence. Patient portals enable patient to look at their charts and imaging results, but also to make future appointments and communicate with their doctor. In countries where electronic health records are installed, the use of such portals is rapidly increasing [29]. Empowering patients with their own health data can cause some anxiety, but it also improves health education and awareness [30]. Tele-consultations and monitoring are more advance digital technique to replace current out-patient clinic visits. In situations where patients have to travel 2 h to talk 15 min to their doctor a video consult is often preferred. Even for new patient contacts, the use of video consultation seems feasible [31].

The current review has some limitations. First, we were only able to include English studies from three databases and may have missed important studies that were not published in the included journals. Second, only practical and logistic properties of the tools were described. Clinometric properties (effectiveness and safety) were not formally studied. It was not possible to perform a meta-analysis due to the limited number of randomized controlled trials and the heterogeneity of their designs and purpose. Third, there are application domains in headache therapy that have not been fully implemented or researched and are therefore underrepresented or even missing in this review. Hence, we would encourage more research and implementation effort in understanding patient use of online information resources for their headache complaints, symptom checkers, digital peer support, remote tele monitoring, the collection and analysis of patient reported outcome metrics (PROMs) in therapy and the use of wearables in collecting data on disease burden and lifestyle.

Future research regarding other digital health tool categories than dairies and behavioral/therapeutical treatment is needed, namely tele-consultations, patient portals, telemonitoring including medication adherence, online information resources, symptom checkers, digital peer support, telemonitoring including proms and telemonitoring including wearables. This will fill the gap in literature regarding digital health tools for headache patients. Next to that, effectiveness of all types of digital health tool categories should be examined. This was not included in most studies, while it seems to be an important aspect because ineffective tools can lead to discrepant and misleading claims, and insufficient quality of the tools [32]. We recommend that health professionals in the headache field, such as general practitioners and neurologists, get inspired by scientific research about digital health tools in other fields than headache. These tools may have a different focus, but the processes and goals behind these tools will be comparable to headache tools.

In summary, many digital health tools for headache patients regarding diaries and behavioral/therapeutical treatment are described in scientific research. However, much scientific knowledge with regard to other categories is still missing. To be more able to ensure quality of digital health tools for headache patients, more knowledge is needed. This mainly relates to tele-consultations, patient portals, telemonitoring including medication adherence, online information resources, symptom checkers, digital peer support, telemonitoring including proms, and telemonitoring including wearables. Without doubt, electronic headache diaries are key to help clarifying diagnosis, headache burden, medication use and therapy response. However, patients are struggling to keep their diaries in a continued and systematic way. Simplification of headache diaries needs to lower data entry burden for patients helping to improve diary compliance.

## Key findings

- Earlier scientific research regarding eHealth for headache patients mainly described electronic diaries and behavioral or therapeutical treatments. Future eHealth studies should focus on the benefits and clinometric properties of these tools. Outcome measures preferably include validated headache and migraine outcome parameters to increase generalizability across studies.
- More knowledge about tele-consultations, patient portals, telemonitoring including medication adherence, online information resources, symptom checkers, digital peer support, telemonitoring including proms, and telemonitoring including wearables is needed.

## Availability of data and material

The data that support the findings of this study are available on request from the corresponding author.
